# The Mediating Role of Professional Quality of Life in the Association Between Structural Empowerment and Transition Among Newly Hired Nurses Educated During the COVID-19 Pandemic

**DOI:** 10.3390/healthcare13172204

**Published:** 2025-09-03

**Authors:** Rawaih Falatah, Nahlah Yahya Beati

**Affiliations:** 1 Nursing Administration and Education Department, College of Nursing, King Saud University, Riyadh 11421, Saudi Arabia; 2 Ministry of Health, Riyadh 12382, Saudi Arabia; nbeati@moh.gov.sa

**Keywords:** newly hired nurses, nursing, Saudi Arabia, nurse education, transition period, structural empowerment, professional quality of life, COVID-19

## Abstract

Background/Objectives: Existing research has highlighted the stress associated with the transition from student to practitioner among newly hired nurses, often resulting in diminished professional quality of life (ProQOL). However, there remains a dearth of understanding regarding the impact of the teaching methods during the COVID-19 pandemic on this transition period. This study aims to test a model assessing the mediating role of ProQOL in the association between structural empowerment and successful transition among newly hired nurses who underwent education during the COVID-19 pandemic. Methods: This study utilized a cross-sectional correlational design and was conducted in two university hospitals and four government hospitals in Saudi Arabia. The study sample was selected using purposive sampling. The Casey–Fink Graduate Nurse Experience Survey, the Arabic version of the ProQOL version 5, and the Conditions for Workplace Effectiveness Questionnaire Second Arabic version were used in the study. Data were analyzed using the Statistical Package for Social Sciences (SPSS) Version 28.0.1.1. The model was examined using Hayes’ process macro. Results: Structural empowerment significantly predicts successful transitions, both directly and indirectly through its impact on ProQOL. Conclusions: Nurse managers should employ optimal strategies and innovative structures within orientation programs to effectively facilitate the transition of Saudi graduate nurses. Moreover, nursing leaders and policymakers should leverage the increased attention garnered during the COVID-19 pandemic to enhance structural empowerment among newly hired nurses, thereby improving their transition and overall well-being. Structural empowerment was a direct and indirect predictor of successful transitions.

## 1. Introduction

Transitioning from education and training to professional independent practice is a stressful and time-consuming period for newly graduated nurses, often causing transition shock [1,2,3]. Several challenges are common during this transition, including anxiety, poor self-confidence, and being overwhelmed, leading to unsuccessful transitions, poor clinical belonging, and turnover [1,2]. Coping practices, such as internships, mentorships, preceptorships, and residency programs, can help with the transition [3,4], but these challenges remain common among new nurses [5,6,7]. Researchers continue to assess the factors that influence this period using tools such as the Casey–Fink Graduate Nurse Experience Survey (CFGNES) [8,9]. However, the transition of nurses educated during the COVID-19 pandemic requires further exploration [10].

COVID-19 disrupted systems globally, including higher education and healthcare [11,12]. Students worldwide experience fear, anxiety, poor concentration, social isolation, and technical and financial issues due to the use of virtual learning [13]. In response, nursing programs adopted contingency plans like virtual simulations and online classes to compensate for lost competency through teaching strategies. This shift hindered students’ clinical preparation for nursing practice, reducing competency and readiness when clinical learning outcomes were not achieved [14]. These conditions likely affected graduates’ transition to practice, lowered job satisfaction, and increased the risk of secondary traumatic stress and burnout. It has also been suggested that increasing structural empowerment may help counter these effects [15].

Healthcare organizations differ in their available resources and budgets for support programs and protocols for graduate nurses, potentially inhibiting a successful transition if not applied effectively. Kanter’s theory of structural empowerment [16] asserts that employees with access to information, resources, and internal and external support are empowered to achieve goals and work efficiently. Despite a dearth of studies relating this to the transition of new graduate nurses [15,17], numerous studies support Kanter’s theory. Such studies find a significant relationship between structural empowerment, job satisfaction, job commitment, and reduced turnover in the general nursing population [18,19].

Job satisfaction is a sense of fulfillment when an individual is aligned with the tenets of their occupation, resulting in a seamless and successful transition [1]. Job satisfaction of new nurses correlates with both internal and external factors. La and Yun [20] stated that anger traits in new nurses are positively associated with poor job satisfaction, whereas anger control is positively associated with job satisfaction. During the transition period, newly graduated nurses rely on respectful and constructive feedback from their preceptors or seniors, demonstrating the potential to increase job satisfaction and reduce turnover [3]. Charette et al. [4] found that structured transition programs aim to improve satisfaction in new nurses. Finally, a supportive work environment and structural empowerment were found to improve job satisfaction and professional quality of life (ProQOL) [21,22,23,24].

ProQOL reflects both positive and negative feelings of those in helping professions. Compassion satisfaction (CS) is positive, whereas compassion fatigue (CF) is negative, leading to burnout and secondary traumatic stress [25]. Numerous studies have examined the differences in ProQOL among new nurses. The average ProQOL ranges between 31.85 and 37.86 for CS, 21.3 and 27.9 for burnout, and 23.1 and 27.17 for secondary traumatic stress [5,6,26,27]. Predictors of new nurses’ ProQOL include transition shock, empathy, resilience, coping style [6], job satisfaction, hours of sleep per day, smoking, workplace violence, and working hours [26]. Kim and Shin [1] found that the successful transition of nurses was significantly predicted by structural empowerment. However, this awareness must be addressed among newly hired nurses who were educated during the pandemic. Further investigation is required concerning the mediating factors of this association.

### 1.1. Purpose of the Study

This study investigated the extent to which the effect of structural empowerment on successful transition is mediated by three components of ProQOL among newly hired Saudi nurses educated during the COVID-19 pandemic. The following hypotheses were tested.

**H1:**  *Structural empowerment directly affects successful transition after accounting for mediators among newly employed Saudi nurses educated during the COVID-19 pandemic.*

**H2:**  *Structural empowerment indirectly affects successful transition through CS among newly employed Saudi nurses educated during the COVID-19 pandemic*.

**H3:**  *Structural empowerment indirectly affects successful transition through burnout among newly employed Saudi nurses educated during the COVID-19 pandemic*.

**H4:**  *Structural empowerment indirectly affects successful transition through secondary traumatic stress among newly employed Saudi nurses educated during the COVID-19 pandemic*.

**H5:**  *The total indirect effect of structural empowerment on successful transition through CS, burnout, and secondary traumatic stress is significant among newly employed Saudi nurses educated during the COVID-19 pandemic*.

### 1.2. Theoretical Framework

This study is underpinned by Kanter’s Theory of Structural Empowerment and Bridges’ Transition Theory, which together offer a comprehensive framework for understanding the transition experiences of newly employed nurses. Kanter’s Theory of Structural Empowerment posits that employees are more effective when they have access to information, resources, support, and growth opportunities within their organizations [16]. Structural empowerment has been associated with improved job satisfaction, reduced burnout, and better transition outcomes for new nurses. This study uses Kanter’s model to examine how organizational structures influence the transition process through the mediating role of ProQOL.

Bridges’ Transition Theory complements this by addressing the psychological and developmental aspects of transition. It describes transition as a three-phase process: ending, neutral zone, and new beginning [28]. For new nurses, this includes letting go of the student role, adjusting to workplace realities, and establishing a professional identity. This framework helps explain how personal and emotional responses—CS, burnout, and secondary traumatic stress—may impact the success of their transition.

Together, these theories underpin our hypothesized model, where structural empowerment (organizational-level factor) influences transition outcomes (individual-level process), both directly and indirectly through ProQOL (individual-level experiences).

## 2. Methods

### 2.1. Study Design

This study utilized a descriptive, cross-sectional, correlational design with mediation analysis to examine the relationship between structural empowerment, ProQOL, and transition experiences among newly hired Saudi nurses. Mediation analysis was performed using Hayes’ PROCESS macro (Model 6).

### 2.2. Study Setting

The study was conducted at two university hospitals and four governmental hospitals in Riyadh, Saudi Arabia. The first setting was a teaching hospital providing primary, secondary, and tertiary healthcare services in various clinical specialties with other supporting services; it has a 1189-bed capacity and 1905 nurses. The second setting was a teaching hospital that provides specialized healthcare services such as ophthalmology and Ear, Nose, and Throat. This facility has a 267-bed capacity and 138 nurses. The third setting was a medical city with 1400 beds and 3700 nurses. The fourth setting was a hospital with a 277-bed capacity and 600 staff nurses. The fifth setting was a hospital with a 207-bed capacity and 490 staff nurses. Finally, the sixth setting was the largest advanced medical complex in Saudi Arabia and the Middle East, with a 1200-bed capacity and approximately 2800 nurses. Riyadh was selected for several reasons, notably because it has the highest number of governmental and private undergraduate nursing programs in Saudi Arabia, increasing access to the target sample. Furthermore, Riyadh has the highest number of healthcare facilities, including teaching hospitals, medical cities, specialized hospitals, and general hospitals.

### 2.3. Study Sample

Purposive sampling was used to recruit all the newly employed graduate Saudi nurses from the study setting. Female and male Saudi nurses with less than one year of employment who graduated from 2020 to 2022 were included in the study. Per Benner’s stages of clinical competence, the novice stage is the first 12 months of employment [29]. Non-Saudi nurses, Saudi nurses who graduated before 2020, nurse managers, and nurse aides were excluded to control for variances in the education systems between countries and between pre- and post-COVID-19 nursing education. These criteria helped to minimize confounding factors.

Using G*Power 3.1, the minimum required sample size was 108, assuming a multiple linear regression test with an effect size of 0.15, power of 0.90, significance level of 0.05, and 4 predictors. After applying the inclusion criteria, 180 participants were invited, and 150 were ultimately recruited. Although we only invited the eligible nurses, after reviewing the data, 32 participants did not meet the inclusion criteria as they graduated before 2020 and therefore were excluded. The estimated response rate was 83.33%, with 150 responses from the 180 invitations.

### 2.4. Measurements

The CFGNES, the Arabic version of the ProQOL version 5 (ProQOL-5), and the Conditions for Workplace Effectiveness Questionnaire Second Arabic version (CWEQ-II-AV) were utilized in the study. Socio-demographic data were also collected, including age, gender, specialty, graduation year, hiring year, certification degree, work schedule, orientation period, and number of preceptors.

#### 2.4.1. Casey–Fink Graduate Nurse Experience Survey

This survey was developed in 1999 by Casey and Fink, and revised in 2002 and 2006. It included five factors: support, patient safety, stress, communication, leadership, and professional satisfaction. It is considered a valid and reliable tool with Cronbach’s alpha of 0.89 for the 24 questionnaire items. Subscale reliability coefficients were α = 0.90 for support, α = 0.75 for communication/leadership, α = 0.79 for patient safety, α = 0.71 for stress, and α = 0.83 for professional satisfaction [30]. The factors were measured on a 4-point Likert scale, ranging from 1 = very unlikely to 4 = very likely.

The CFGNES–Revised was translated and culturally adapted into Arabic using the integrated method described by Sidani et al. [31], emphasizing linguistic accuracy and cultural relevance. Two bilingual experts—one clinical and one translation specialist—completed forward translations, which were synthesized into one version. Two other bilingual individuals unfamiliar with the original tool completed back-translations into English. A five-member expert panel, including nursing educators, researchers, and bilingual clinicians, evaluated the translated version for semantic, idiomatic, experiential, and conceptual equivalence. Based on their input, a pre-final pilot was developed and tested with 30 newly graduated nurses to assess clarity, comprehension, and cultural appropriateness. The final Arabic version maintained the psychometric integrity and conceptual meaning of the original instrument. In this study, the CFGNES demonstrated sufficient reliability (α = 0.88); subscale alphas were: communication/leadership α = 0.69, support α = 0.87, patient safety α = 0.69, and professional satisfaction α = 0.70.

#### 2.4.2. The Conditions for Workplace Effectiveness Questionnaire-II-Arabic Version

The CWEQ-II-AV was used to measure structural empowerment and consists of 19 items rated on a 5-point Likert scale (1 = no access to structural empowerment and 5 = a lot of access to structural empowerment). The items were divided into six subscales: opportunity, resources, support, information, formal power (job activity scale; JAS), and informal power (organizational relationship scale; ORS). Each subscale included three items, except for the ORS, which had four. The Global Empowerment (GE) subscale, added to the survey in 1995 as a validation index, includes two items that measure empowerment in the work environment. The total scale’s established reliability coefficient was α = 0.95; subscales ranged from α = 0.83–0.89 [32]. The questionnaire was used previously in the Saudi nursing context [33]. The CWEQ-II-AV demonstrated adequate reliability for the overall questionnaire (α = 0.93) in this study; subscale alphas were opportunity α = 0.89, information α = 0.88, support α = 0.92, resources α = 0.82, JAS α = 0.75, ORS α = 0.81, and GE α = 0.90. Scores of 6–13 indicate low empowerment levels, 14–22 indicate moderate empowerment levels, and 23–30 indicate high empowerment levels.

#### 2.4.3. The Professional Quality of Life Scale Version 5

The Arabic version of the ProQOL-5 measures positive CS and negative CF effects on those who work with individuals who have experienced stressful events. The CF can be divided into two scales: burnout, resulting from exhaustion and depression, and secondary traumatic stress. The scale comprises 30 items rated on a 5-point Likert scale (1 = never, 2 = rarely, 3 = sometimes, 4 = often, and 5 = very often). Cronbach’s alpha for CS, burnout, secondary traumatic stress, and the 30-item survey were 0.90, 0.76, 0.81, and 0.75, respectively [34]. The scale has been used in the Saudi nursing context, e.g., Ageel and Shbeer [35]. In this study, overall reliability was α = 0.84; CS was α = 0.91, burnout was α = 0.62, and secondary traumatic stress was α = 0.83. Scores ≤ 22 indicate low reliability levels, 23–41 indicate moderate reliability, and ≥41 indicate high reliability levels for all three components.

### 2.5. Data Collection

Data were collected through an anonymous online survey. After obtaining institutional review board approval from King Saud University (KSU-HE-22-123) and gaining permission from the selected hospitals, the study’s aim and procedure were explained to the nursing affairs managers of each hospital. Participants received a recruitment message with a brief study description, instructions, and estimated completion time. Data collection occurred in two phases. In phase one, a link to the online survey was distributed to eligible nurses identified by their unit managers. One week later, in phase two, the researcher visited the units to remind participants and collect data from the nurses who needed assistance completing the survey in the initial stage. The researchers used an iPad to facilitate data collection.

### 2.6. Data Analysis

Descriptive and inferential statistics for all items were calculated using IBM Statistical Package for Social Sciences (SPSS) Version 28.0.1.1, widely used in social and health sciences. The Hayes process macro for SPSS was also used to conduct the mediation analyses. Missing data analysis showed no missing variables. Univariate and bivariate analyses were used to describe the sample and explore violations of statistical assumptions. Multivariate analysis tested the study hypotheses using the Hayes process macro [36].

## 3. Results

### 3.1. Socio-Demographic Characteristics of the Participants

The socio-demographic characteristics of the 118 participants are presented in Table 1. Most were female (72.9%, n = 86), with a mean age of 24.68 years (SD = 1.583, range 22–36 years). The graduation years of the participants ranged from 2020 to 2022; 43.2% (n = 51) graduated in 2020. Most held a bachelors degree (94.9%, n = 112). In this sample, 39.8% (n = 47) were employed in 2021 and 60.2% (n = 71) were employed in 2022. Regarding the work patterns, 69.5% (n = 82) rotated between the day and night shifts, 29.7% (n = 35) had only day duty, and 0.8% (n = 1) had only night duty. The length of unit orientation varied, with almost one-third of participants (28.8%, n = 34) in orientation for 9 to 12 weeks. The mean number of primary preceptors during orientation was 2.10 (SD = 1.21), ranging from 0 to 7.

### 3.2. Study Variables

The descriptive statistics of the study variables are presented in Table 2. Three questionnaires were used to measure variables. The overall Casey–Fink scale mean was 69.86 (SD = 9.81). The highest Casey–Fink subscale mean was professional satisfaction (3.21, SD = 0.64), and the lowest was stress (0.23, SD = 0.18). Structural empowerment was measured using the CWEQ-II-AV, with an overall mean of 21.49 (SD = 5.57). The highest CWEQ-II-AV subscale mean was access to opportunity (3.39, SD = 1.15), and the lowest was for the job activities scale (formal power) (2.58, SD = 0.97). ProQOL subscale means were CS 37.86 (SD = 8.27), burnout 26.87 (SD = 6.66), and secondary traumatic stress 24.37 (SD = 7.63).

### 3.3. Correlation Analysis

Pearson correlation coefficients were calculated to examine the relationships among structural empowerment, transition experience, CS, burnout, secondary traumatic stress, scheduled work pattern, orientation length, and the number of preceptors (Table 3). Structural empowerment was positively and significantly correlated with transition experience (r = 0.582, *p* < 0.001) and CS (r = 0.607, *p* < 0.001), and negatively correlated with burnout (r = −0.605, *p* < 0.001) and secondary traumatic stress (r = −0.200, *p* = 0.030). Transition experience was also positively correlated with CS (r = 0.557, *p* < 0.001) and negatively correlated with burnout (r = −0.616, *p* < 0.001) and secondary traumatic stress (r = −0.358, *p* < 0.001). Burnout was positively correlated with secondary traumatic stress (r = 0.421, *p* < 0.001). Among covariates, only the number of preceptors showed a weak positive correlation with secondary traumatic stress (r = 0.184, *p* = 0.046). Other associations among covariates were not statistically significant.

### 3.4. The Direct and Indirect Association Between Structural Empowerment and Transition

**Figure 1:**
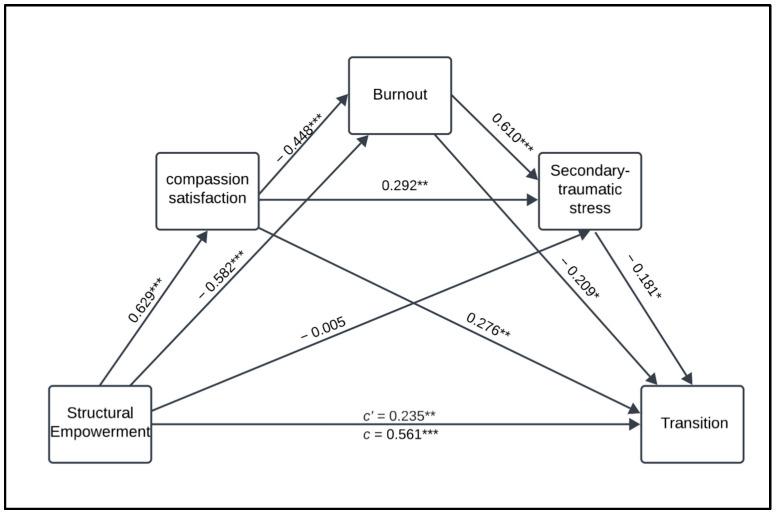
Mediation model illustrating the direct and indirect effect of structural empowerment on transition through profession quality of life components. * *p* ≤ 0.05; ** *p* ≤ 0.01; *** *p* ≤ 0.001.

Hayes’ PROCESS macro Model 6 was used to examine the indirect effect of structural empowerment on the transition experience through three sequential mediators: CS, burnout, and secondary traumatic stress (Figure 1). In this model, structural empowerment was the independent variable, transition was the dependent variable, and scheduled work pattern, orientation length, and number of preceptors were the covariates. The analysis was conducted using SPSS (Version 28.0.1.1.) with the PROCESS macro. We used bootstrapping with 5000 resamples to estimate the confidence intervals for indirect effects and determine their significance. Assumptions of normality, linearity, homoscedasticity, and multicollinearity were assessed before the analysis and met acceptable thresholds. This approach allowed for a robust estimation of direct and indirect relationships among the study variables.

Model fit: The overall model fit was good, with a significant R-squared value (R^2^ = 0.506), indicating that 50.6% of the variance in transition was elucidated by the model.

#### 3.4.1. Direct Effects

The direct effect of structural empowerment on transition was significant (β = 0.235, B = 0.414, SE = 0.162, *p* = 0.012), indicating that structural empowerment has a direct impact on new nurses’ transition, independent of their ProQOL components.

#### 3.4.2. Indirect Effects

Mediator 1 (CS): The indirect effect of structural empowerment on the transition through CS was significant (β = 0.174, B = 0.306, SE = 0.069, 95% CI [0.0239, 0.302]). This finding suggests that CS partially mediates the relationship between structural empowerment and the transition process.

Mediator 2 (burnout): The indirect effect of structural empowerment on the transition through burnout was insignificant (β = 0.064, B = 0.112, SE = 0.045, 95% CI [−0.007, 0.167]), indicating no mediation effect.

Mediator 3 (secondary traumatic stress): The indirect effect of structural empowerment on the transition through secondary traumatic stress was also insignificant (β = 0.001, B = 0.002, SE = 0.023, 95% CI [−0.048, 0.049]), indicating no mediation effect.

#### 3.4.3. Total Indirect Effect

The total indirect effect of structural empowerment on transition through all three mediators was significant (β = 0.561, B = 0.987, *p* < 0.001, SE = 0.136, 95% CI [0.718, 1.257]), indicating that the mediators collectively account for a substantial portion of the relationship between structural empowerment and the transition process.

## 4. Discussion

This study surveyed 118 nurses to examine the direct and indirect effects of structural empowerment on the transition of new nurses through the three components of ProQOL. The transition score was slightly higher than that reported by Kim and Shin [1]. Additionally, the transition score in the current sample (M = 69.86, SD = 9.81) reflects the total CFGNES score. For comparison, Casey et al. [30] reported a lower mean total score (M = 44.67, SD = 7.51) among nursing students completing their senior practicum experience [Ref]. While differences in sample and setting limit direct comparisons, these figures suggest variation in transition readiness across different populations and contexts. Our sample’s higher mean score may reflect differences in education, clinical experience, or environmental factors. However, no formal statistical comparison was conducted, and these differences should be interpreted with caution. The Casey–Fink score from both the literature and the current study suggests that COVID-19-related educational changes did not affect students’ transition to nursing. This might be attributed to nursing students’ high resilience, coping skills, and strategies [37]. Finally, while virtual simulations and online classes were implemented during the pandemic, we did not directly measure their impact on transition outcomes. Therefore, no causal inferences can be made regarding their effect on the observed transition scores in this study.

Organizations are crucial in transitioning new nurses through factors such as structural empowerment. This study measured structural empowerment using CWEQ-II-AV, with an overall mean of 21.49, indicating moderate structural empowerment. This finding is slightly higher than, but comparable to, the findings among a sample of new Jordanian nurses, with both studies reporting the highest subscale as access to opportunity (M = 3.39, SD = 1.15) and the lowest being the job activities scale of “formal power” [32]. Similarly, Sarıköse and Çelik [23] reported moderate structural empowerment among a sample of newly employed nurses in Turkey. Using the 12-item CWEQ-II, Boamah and Laschinger [15] reported that the mean overall structural empowerment of a sample of newly employed nurses was 13.03, also indicating moderate empowerment.

The observed moderate levels of structural empowerment might be attributed to increased awareness regarding the importance of nurses’ roles in patient care, especially post-pandemic [38]. Additionally, the growing demand for healthcare accreditation and standards, such as Magnet Recognition, which emphasizes nurse engagement and empowerment, may contribute to this level of empowerment. Therefore, it can be argued that new nurses who were educated before and after the pandemic were employed in organizations with structures that provide them with moderate empowerment. This level of structural empowerment may enhance new nurses’ ProQOL and improve their transition experiences.

This study measured ProQOL using the ProQOL scale, with the sample scoring moderately on all three components. Similarly, Algamdi [34] found that 334 Saudi nurses working in Tabuk City, Saudi Arabia, had moderate levels of burnout (68.3%) and secondary traumatic stress (71.3%); however, they had a high level of CS (57.5%). Moderate levels of burnout, CS, and secondary traumatic stress have been observed among newly graduated Chinese nurses [5,6,27]. These findings suggest that most newly employed nurses, both pre- and post-pandemic, have moderate levels of CS, burnout, and secondary traumatic stress. Moderate levels of CF components can be attributed to the demanding nature of the nursing profession and frequent exposure to traumatic situations, particularly during the pandemic [26].

In this study, the transition of new nurses was significantly predicted by structural empowerment; the three ProQOL components slightly moderated this relationship. CS was a significant mediator of the indirect relationship between structural empowerment and transition. Although CF components did not significantly mediate the relationship between structural empowerment and transition, the overall model was significant and explained 50% of the change in the transition of new nurses. Zeng et al. [27] found that secondary traumatic stress and CS predict post-traumatic growth; hence, they might facilitate the transition. Therefore, based on the current study and literature, it can be argued that enhancing structural empowerment and ProQOL is important for new nurses’ transition and retention [1].

Although CS significantly mediated the relationship between structural empowerment and transition, burnout and secondary traumatic stress did not show significant indirect effects in the model. This may indicate that while positive aspects of ProQOL, such as CS, play a meaningful role in shaping new nurses’ transition outcomes [27], the negative aspects, such as burnout and secondary traumatic stress, may exert less direct influence, or their effects may be moderated by other contextual or individual factors not examined in this model [2,33]. The lack of significance in these pathways may also suggest that, while statistically significant, the model only partially explains the complexity of new nurses’ transition experiences. Unmeasured variables—like resilience, coping mechanisms, or perceived organizational support—might better capture the indirect effects of stress-related constructs [16,31]. Future research should consider integrating these psychosocial and organizational variables to develop a more comprehensive transition model among newly employed nurses. Finally, there is no evidence that the teaching strategies during the pandemic negatively impacted nursing education outcomes or new nurses’ transitions.

Although the model explained 50.6% of the variance in new nurses’ transition, a substantial proportion (49.4%) remains unexplained. This suggests that additional unmeasured variables—like workplace culture, leadership support, individual resilience, or interprofessional collaboration—may influence transition outcomes. These contextual and psychosocial factors were not assessed in this study and should be considered in future research. Furthermore, while model fit indices such as RMSEA and CFI are commonly used in structural equation modeling, they were not reported here as the mediation analysis was conducted using the PROCESS Macro in SPSS, which uses OLS regression and does not produce global fit indices. This methodological limitation is acknowledged, and future studies employing path or SEM approaches could provide a more comprehensive assessment of model fit.

### Limitations

This study has some limitations. First, the cross-sectional correlational design prevents testing of the causal relationships between the study variables. Second, nonprobability sampling limits the generalizability of the findings to all newly hired nurses. Furthermore, the study’s sample size was relatively small, and the involved hospitals were all located in one city, which may affect generalizability to other geographical locations and cultures; therefore, the results should be verified in different locations and cultures. Future research should use an experimental and longitudinal approach to build on these findings. Finally, the use of self-reported scales may have introduced bias.

The current study’s findings provide valuable data regarding the predictors of the transition period among newly graduated nurses educated during COVID-19. Based on these results, it can be argued that the strategies used, including online classes and virtual simulation, did not impact the perceptions of the new nurses concerning structural empowerment and ProQOL. Therefore, nursing programs worldwide should leverage the opportunities and lessons learned during COVID-19 to increase admissions to nursing programs and reduce nursing shortages. Senior students must be prepared for their professional roles, workplace realities, and transition challenges. Nursing leaders and policymakers should consider adapting work environments and implementing programs that support independent practice and reduce the micromanagement of new nurses.

## 5. Conclusions

To our knowledge, this study is the first to apply a model that explores both the direct and indirect effects of structural empowerment on the transition of newly employed Saudi nurses, with ProQOL as a mediator. The results confirm that structural empowerment significantly predicts transition outcomes and that CS partially mediates this relationship. The model explained 50% of the variance in transition, highlighting the substantial role of organizational factors in shaping early professional experiences.

Notably, no differences were observed in transition scores among nurses educated during the COVID-19 pandemic compared to those trained in traditional settings. This suggests that innovative teaching modalities—such as online learning and virtual simulation—may have mitigated the potential negative impact of disrupted clinical experiences. These findings reinforce the value of flexible, technology-driven education approaches in preparing nursing graduates for clinical practice.

Given these insights, several targeted strategies are recommended. Nursing education programs should continue to adopt and refine virtual simulation and online modalities, especially in regions with limited clinical placements. Healthcare organizations should prioritize structural empowerment by embedding shared governance models, fostering transparent communication channels, and creating accessible professional development pathways for novice nurses. Establishing formal mentorship programs and offering early career coaching can also enhance CS and reduce the risk of burnout.

Moreover, healthcare policymakers and hospital leadership should leverage the increased recognition of the nursing profession gained during the pandemic to institutionalize empowerment frameworks. This can support the smoother nursing transitions and contribute to higher job satisfaction, reduced turnover, and better patient care outcomes. In summary, this study provides a foundation for educational and organizational interventions to strengthen the transition experiences of new nurses within Saudi Arabia and similar healthcare contexts.

## Figures and Tables

**Table 1:** Socio-demographic characteristics of participants (N = 118).

Range	SD	Mean	Characteristic
22–36	1.58	24.68	Age
0–7	1.21	2.1	Number of primary preceptors
Percentage (%)		Frequency (n)	Characteristic
			Gender
27.1		32	Male
72.9		86	Female
			Graduation year
43.2		51	2020
41.5		49	2021
15.3		18	2022
			Education level
5.1		6	Diploma
94.9		112	Bachelor’s degree
			Year hired
39.8		47	2021
60.2		71	2022
			Scheduled work pattern
69.5		82	Rotating days/nights
29.7		35	Only days
0.8		1	Only nights
			Duration of unit orientation
15.3		18	Still ongoing
27.1		32	≤8 weeks
28.8		34	9–12 weeks
11		13	13–16 weeks
5.1		6	17–23 weeks
12.7		15	≥24 weeks

**Table 2:** Study variable descriptive statistics (N = 118).

Variable	Mean	SD	Range	Skewness		Kurtosis	
				Statistics	Std. Error	Statistics	Std. Error
Casey–Fink factors	3.11	0.578	4.00–1.33	−0.510	0.223	0.201	0.442
Casey–Fink organizing and prioritizing factors	2.82	0.554	4.00–1.00	−0.225	0.223	0.688	0.442
Casey–Fink stress factors	0.23	0.18	0.67–0.00	0.406	0.309	−0.70	0.61
Casey–Fink communication/leadership factors	3.02	0.48	4.00–1.83	0.286	0.223	−0.227	0.442
Casey–Fink professional Satisfaction factors	3.21	0.64	4.00–1.67	−0.384	0.223	−0.68	0.442
Casey–Fink total	69.86	9.81	92.00–43.00	0.079	0.223	0.127	0.442
Access to opportunity	3.39	1.15	5.00–1.00	−0.339	0.223	−0.668	0.442
Access to information	2.74	1.12	5.00–1.00	−0.103	0.223	−0.754	0.442
Access to support	3.21	1.13	5.00–1.00	−0.310	0.223	−0.454	0.442
Access to resources	3.15	0.97	5.00–1.00	−0.154	0.223	−0.106	0.442
Job activities scale (formal power)	2.58	0.93	5.00–1.00	0.027	0.223	−0.260	0.442
Organizational relationships scale (informal power)	3.37	0.97	5.00–1.00	−0.353	0.223	−0.171	0.442
Global empowerment	3.05	1.21	5.00–1.00	−0.143	0.223	−0.919	0.442
Total structural empowerment	21.49	5.57	32.83–7.00	−0.431	0.223	0.015	0.442
Compassion satisfaction	37.86	8.27	50.00–10.00	−0.892	0.223	0.970	0.442
Burnout	26.87	6.66	46.00–1300	0.323	0.223	0.183	0.442
Post-traumatic stress	24.37	7.63	44.00–10.00	0.239	0.223	−0.431	0.442

**Table 3:** Correlation analysis result.

	1	2	3	4	5	6	7	8
1. Structural empowerment								
2. Transition experience	0.582 **							
3. Compassion satisfaction	0.607 **	0.557 **						
4. Burnout	−0.605 **	−0.616 **	−0.631 **					
5. Post-traumatic stress	−0.200 *	−0.358 **	−0.077	0.421 **				
6. Scheduled work pattern	−0.153	−0.149	−0.057	0.146	0.130			
7. Length of unit orientation	0.005	0.052	−0.097	0.044	−0.034	0.086		
8. Number of primary preceptors	−0.138	−0.157	0.018	0.179	0.184 *	0.104	0.134	

* *p* < 0.05, ** *p* < 0.01.

## Data Availability

The data presented in this study are available on request from the corresponding author.
